# Intubating Stylets in the Emergency Department: A Video Review of First-pass Success and Time

**DOI:** 10.5811/westjem.47204

**Published:** 2025-10-04

**Authors:** Raymond Che, Niaman Nazir, Ali Badar, Anchitha Honnur, Mark Newton, Abdel-Rahman Mohammed Samour, Tala Samour, Dane Clutter, Andrew Pirotte

**Affiliations:** * University of Kansas Health System and University of Kansas Medical Center, Department of Emergency Medicine, Kansas City, Kansas; † University of Kansas Health System and University of Kansas Medical Center, Department of Population Health, Kansas City, Kansas; ‡ University of Kansas School of Medicine, Kansas City, Kansas; § University of Kansas, Lawrence, Kansas; || Al-Balqa Applied University, Faculty of Medicine, Salt, Jordan; ** Kansas City University College of Osteopathic Medicine, Kansas City, Missouri

## Abstract

**Introduction:**

Effective airway management is critical for optimal patient outcomes in the emergency department (ED). Additionally, airway management is significantly influenced by the clinician’s selection of equipment, specifically the choice of intubating stylet. Also of note, the duration of intubation (time to intubate) impacts overall success. The choice of intubation device may influence first-pass success and intubation times. In this study we evaluated equipment trends for first-pass success and intubation duration. We collected data by reviewing a video database of recorded airways. Three commonly used intubating stylets were reviewed: the hyperangulated stylet; bougie (Eschmann stylet); and malleable stylet.

**Methods:**

In this retrospective observational study, we reviewed 615 intubation videos. These videos were recorded via video laryngoscopy at the University of Kansas Medical Center and The University of Kansas Health System between February 2019–January 2022. We recorded device type, number of intubation attempts, and time to successful intubation (time from entry of laryngoscope blade to passage of endotracheal tube through vocal cords). We included and analyzed 575 intubations for first-pass success, while a random subset of 70 intubations was used to evaluate intubation times. We also conducted a survey to query current faculty and resident physicians regarding their preference for intubation modality.

**Results:**

Among 575 intubations, the bougie (Eschmann stylet) was used in 47.1% of cases, the malleable stylet in 27.3%, and the hyperangulated (also known as “rigid” or “angular”) stylet in 25.6%. Overall first-pass success was 91.3%. The malleable stylet showed the highest success rate (94.9%), followed by the hyperangulated stylet (93.2%), and the bougie (88.2%) (χ^2^ = 6.53, P = .04). In a separate analysis of 70 cases, the median intubation time was 35.5 seconds. For intubation time, we found a significant difference between the three modalities (χ^2^ = 8.2019, P = .02), with pairwise differences between bougie vs malleable stylet (P = .01) and bougie vs hyperangulated stylet (P = .02), but not between hyperangulated and malleable stylets (P = .62). Bougie-assisted intubations had the highest median time of 40.5 seconds (mean 49.15 +/− 23.1) compared to malleable stylet 31 seconds (mean 33.8 +/− 16.4) and hyperangulated 31 seconds (mean 33.6 +/− 11). A survey of 52 physicians showed that 55.8% preferred the malleable stylet, 19.2% preferred the hyperangulated stylet, and 25% preferred the bougie.

**Conclusion:**

The malleable stylet demonstrated the highest first-pass success rate and the most consistent intubation times, while the bougie had the longest times and lowest success rate in our ED. Physician preferences also favored the malleable stylet. First-pass success rates and intubation times vary depending on an institution’s familiarity with specific devices and the clinician’s preference. These factors should be considered when selecting intubation equipment to optimize airway management outcomes or facilitate training.

## INTRODUCTION

Effective and efficient emergency airway management is a keystone of success in the care of critically ill patients in the emergency department (ED). First-pass success during intubation is a meaningful metric in the ED, where rapid and effective airway management is essential. Each additional intubation attempt (after the first attempt) increases the risk of adverse events, including witnessed aspiration, oxygen desaturation, esophageal intubation, hypotension, dysrhythmia, and cardiac arrest.^1^ First-pass success rates vary based on several factors including the clinician’s experience,^2^–^3^ patient characteristics such as restricted neck extension or mouth opening,^3^ or choice of airway device.^4^

Commonly used devices to facilitate endotracheal intubation include the following:

1. Malleable stylet: a semi-flexible, plastic-coated metal device used to give shape to an endotracheal tube
2. Bougie (Eschmann stylet): a narrow, soft plastic introducer placed into the trachea first, over which the endotracheal tube is advanced
3. Hyperangulated (or rigid) stylet: a rigid metal introducer that maintains its fixed shape throughout the intubation process

Of note, recent expert recommendations suggest routine use of the bougie over the malleable stylet, while the rigid stylet is preferred when hyperangulated laryngoscope blades are used.^5^

Several studies have compared the effectiveness of different airway introducers across various clinical settings. These studies have served to evaluate first-pass success rates, with variable results. Several articles reported a significant increase in first-pass success with the use of a bougie.^4^,^6^–^9^ On the other hand, there have also been reports of no statistically significant difference between bougie and malleable stylets.^10^–^12^ Additionally, studies evaluating the difference in first-pass success when using a malleable stylet vs a hyperangulated stylet have had varying results.^13^–^14^ Notably, two prior observational studies assessing airway introducers were conducted in the prehospital setting, limiting their generalizability to ED intubations.^7^–^9^ In this study we aimed to leverage the University of Kansas Medical Center and The University of Kansas Health System airway video database (AVD) to contribute to the existing literature. By evaluating trends in first-pass success rates across all three devices, this study provides a real-world dataset of video-recorded intubations conducted in the ED.

## METHODS

### Design, Setting, and Population

This was a retrospective observational study conducted at the University of Kansas Medical Center and The University of Kansas Health System Emergency Department, an academic, tertiary-care facility and Level I trauma center with approximately 85,000 visits annually. We analyzed 615 video-recorded intubations collected between February 6, 2019–January 18, 2022, from the AVD. The primary objective was to evaluate intubation success rates for each device type and the duration of intubation attempts. A secondary objective was to assess physician device preferences via a survey.

**Population Health Research Capsule**

What do we already know about this issue?

*Emerging data supports the bougie as superior for first-pass success, with expert consensus recommending it as a first-line device in endotracheal intubation*.

What was the research question?

*Which intubating stylet used in our ED is associated with the highest first-pass success rate?*

What was the major finding of the study?

*Malleable stylet had the greatest first-pass success rate (94.9%, CI .92–.98) vs bougie (88.2%, CI .84–.92), P = .04*.

How does this improve population health?

*Optimizing stylet choice improves first-pass success and reduces complications, enhancing airway outcomes and care quality in ED patients*.

### Data Source: Airway Video Database (AVD)

The AVD is an independent, pre-existing, and ongoing collection of video laryngoscopies captured using GlideScope (Verathon, Inc, Bothell, WA) and C-MAC (Karl Storz SE & Co. KG, Tuttlingen, Germany) devices. It serves as a free, open-access educational resource designed for airway education. We have no conflict of interest regarding the devices used. All patient identifying information, such as demographics, medical history, procedural circumstances, and performing clinician details, are removed to ensure confidentiality. The videos have no link to the electronic health record and cannot be traced to the patient. For organizational and repeatability purposes, each video is assigned a unique, arbitrary identifier composed of a patient age and a chief complaint.

### Inclusion and Exclusion Criteria

We analyzed all recorded attempted intubations available in the AVD during the study period. Videos were excluded if data were missing, if the intubation involved multiple attempts using different devices, or if the intubation was a bimodal airway (e.g., bronchoscopy assisted). For intubation duration, we excluded any video that contained multiple attempts or had unclear visualization of the beginning or end of the intubation process. However, during data abstraction of intubation duration, no selected videos met exclusion due to poor visualization.[Fig f1-wjem-26-1374]

### Abstractor Training and Blinding

One trained abstractor manually reviewed all videos and compiled data into a standardized spreadsheet. Before data collection began, the abstractor was oriented to the AVD interface and trained on how to enter data consistently. For the time-interval measurements, additional abstractors assisted in reviewing a subset of intubations, although most reviews were performed by a single individual. All reviewers were trained to identify the procedural start and end points, defined as the insertion of the laryngoscope blade and the passage of the endotracheal tube through the vocal cords, using embedded video timestamps. Abstractors were not blinded to the study’s objectives; however, the original spreadsheet used for data abstraction had been created prior to the study’s conception and remained unchanged to minimize bias.

### Data Abstraction and Collection

Each video was assigned a unique numerical identifier from 1 to 615. Data were manually abstracted into a standardized spreadsheet, including the intubation device used and the number of attempts. For time-based analysis, we randomly selected 70 intubations using a computer-generated number list. If a selected video met exclusion criteria, a new random video was chosen. Duration was defined as the time in seconds from insertion of the laryngoscope blade to passage of the endotracheal tube through the vocal cords, determined using timestamps within the video.

#### Survey on Device Preference

To assess preferred intubation methods, we conducted a survey of emergency department faculty and residents. The survey included questions about device preferences. A total of 52 of 77 invited participants (67.5%) completed the survey. Participation was voluntary, and responses were anonymous.

#### Ethical Considerations

As this study involved a secondary retrospective analysis of de-identified video data, it was reviewed and deemed to be exempt by our institutional review board (IRB).

We adhered to the criteria set forth by Worster et al^15^ as follows: 1) Abstractors were trained in navigating and using the AVD; 2) inclusion and exclusion criteria were clearly defined; 3) study variables were defined; 4) data were collected using standardized spreadsheets; 5) abstractor performance was periodically reviewed by the principal investigator to ensure consistency and accuracy within data entries; 6) abstractors were not blinded to the study objectives, although the original data abstraction spreadsheet was created prior to study conception; (7) interobserver reliability is addressed in the limitations section; (8) formal interobserver reliability testing was not conducted due to the small number of abstractors and the fact that a single abstractor completed the majority, if not the entirety, of each dataset; 9) the AVD data source was thoroughly described; 10) the sampling method was clearly outlined; 11) any video missing predefined variables were removed; and 12) the study was reviewed by the IRB.

### Statistical Analysis

We performed data management and statistical analyses using SAS software v9.4 (SAS Institute Inc., Cary, NC). We summarized categorical variables with percentages, and continuous variables by means and medians. The chi-square test was used to make global comparisons of categorical variables across the three device types. As the time data were skewed, we used a non-parametric Kruskal-Wallis test to make global comparisons of time across the three device types. Similarly, pairwise comparisons of time across devices were made using the Wilcoxon two-sample test. Two-sided *P*-values < .05 were considered statistically significant.

## RESULTS

We analyzed 615 intubations. Twenty-five cases (4.0%) either had missing data or were excluded as multiple modalities had been used in addition to multiple attempts. Another 15 (2.4%) cases that used a bimodal technique (laryngoscope plus bronchoscope on split screen) were excluded. Among the 575 intubations included in this study, the bougie was the most frequently used device, accounting for 271 (47.13%) cases. The malleable stylet was used in 157 (27.30%) intubations, and the hyperangulated stylet in 147 (25.57%). The bougie was selected nearly twice as often as the hyperangulated or malleable stylet.[Table t1-wjem-26-1374]

Among 575 intubations analyzed, first-pass success rates varied by stylet type (χ^2^ = 6.53, *P* = .04). The malleable stylet had the highest first-pass success rate (94.9%), followed by the hyperangulated stylet (93.2%) and the bougie (88.2%). The bougie was associated with the highest proportion of multiple attempts (11.8%), compared to 6.8% for the hyperangulated stylet and 5.1% for the malleable stylet. The overall total first-pass success rate was 91.3%

We selected 70 samples at random from the database, and the time of intubation was manually measured. Five cases had multiple attempts. These were excluded and replaced with another randomly generated case for the airway from the cases included in the airway database for a total sample size of 70. The median intubation time of the sample was 35.5 seconds.

Bougie-assisted intubations show the longest median time of 40.5 seconds (mean 49.15 +/− 23.1) and highest variability, with a maximum time of 93 seconds. Intubation time with the malleable stylet was 31 seconds (mean 33.8 +/− 16.4), and hyperangulated was 31 seconds (mean 33.6 +/− 11). Comparison of the distribution of intubation time intervals between all three modalities revealed a statistically significant difference between these groups (χ^2^ = 8.2019, *P* = .02). The pairwise comparisons of intubation time between devices demonstrated a statistically significant difference between bougie vs malleable stylet (*P* = .01) and bougie vs hyperangulated stylet (*P* = .02). However, there was not a statistically significant difference between hyperangulated stylet vs malleable stylet (*P* = .62).

### Survey of Clinician Preferences

To further explain such differences, we conducted a survey that sampled 77 total attending physicians, fellow physicians, and resident physicians regarding their preference for intubation modalities. This cohort consisted of most of the physicians composing the sample dataset. A total of 52 results were collected from the 77 who were surveyed. Results showed that 55.8% of responders preferred the malleable (gray) stylet, 19.2% the hyperangulated stylet, and 25% the bougie (Eschmann stylet).[Table t2-wjem-26-1374]

## DISCUSSION

There is conflicting research regarding the effect of stylet modalities on successful intubations.^4^,^6^–^12^ Some of this research focused on non-hospital settings, which limited reproducibility in ED intubations. This highlights the need for further analysis of intubation modalities in the ED setting. In this study we used AVD of the University of Kansas Medical Center and The University of Kansas Health System to stratify the effectiveness of stylet modalities through real-world emergent intubations.

At our institution, the bougie (Eschmann stylet) was the most commonly used stylet modality during endotracheal intubation. Despite this finding, our results indicate that the malleable stylet was associated with the highest rate of first-pass success, followed by the hyperangulated stylet. Both modalities outperformed the bougie, which demonstrated statistically significantly lower rates of first-pass success. The malleable and hyperangulated stylets achieved the fastest successful intubation times, with similar results, while the bougie required the longest time for successful intubation.

There has been previous expert recommendation for the routine use of a bougie which is supported by evidence of superior first-pass success in a variety of clinical scenarios.^4^–^9^ In this study we present a possible advantage of the malleable stylet over the bougie stylet in both first-pass success and intubation time. Institution-specific training, experience, familiarity with a device, and preference may contribute to higher first-pass success rates and reduced intubation times. It is important to acknowledge that many other variables may affect both intubation success and timing. For instance, laryngoscope selection may have an equal (or even greater) impact on stylet choice (e.g., in a cardiac arrest patient, the hyperangulated blade may be selected). This selection typically necessitates the use of the rigid/hyperangulated stylet and precludes selection of malleable stylet or bougie, as these stylets are not well suited for the geometry of a hyperangulated blade. In such cases, the laryngoscope dictates the intubating adjunct, rather than the stylet being chosen independently. This relationship suggests that device selection and clinical context may significantly influence intubation outcomes. Therefore, it may be important to consider not only the stylet itself but the broader clinical context when interpreting first-pass success and intubation time. We were unable to fully account for the interplay between laryngoscope and stylet selection or the clinical context in which each device is used, which represents an interesting and promising avenue for future research.

We found notable discrepancies when correlating data regarding clinician preferences (via the survey) and trends seen in the AVD. While the survey data showed that the malleable stylet was the most preferred device, a review of the AVD showed that the bougie was the most employed device. This mismatch may reflect an institutional effort to promote bougie use in response to emerging literature supporting its effectiveness. However, the survey results suggest that clinicians may still feel less comfortable or experienced with bougie-assisted intubation. Despite this variance, the data do align with the malleable stylet as the most effective intubation modality in this ED.

## LIMITATIONS

This study has several limitations. Because the AVD is de-identified for privacy concerns, the age, sex, presenting complaint, or comorbidities of the patient could not be correlated to the intubation. And given the single-center design of this study, the generalizability and external validity of the findings may be limited. Additionally, we did not formally address interobserver variability among the abstractors measuring intubation duration. While all abstractors used the same operational definitions for procedural start and end points, subtle differences in interpretation may have introduced variability, potentially affecting the precision of duration estimates. Furthermore, abstractors for the intubation duration dataset were not blinded to the study objectives, which could have introduced bias, particularly in the interpretation of time-based outcomes. However, the original abstraction spreadsheet had been created prior to the study’s conception and remained unchanged, which may have helped mitigate this bias.

These results may also have been biased by clinician preference for intubation modalities. For example, although the bougie stylet was the most common form of stylet used in the AVD, this was not the subjectively preferred method of intubation based on the physician survey. Another confounding variable was the motivation behind equipment selection, which could not be fully assessed due to the de-identified nature of the database. For example, a bougie may be selected for anticipated difficult airways. This would mean the difficulty of the airway itself, rather than the modality, could influence success rates and could be a potential direction for future research. User familiarity and experience level may also impact outcomes. Although our study favored the malleable stylet, there were numerous examples in the AVD where the bougie achieved rapid first-pass success or was used effectively as a rescue device when the initial modality had failed. Thus, these findings should be interpreted cautiously.

We analyzed intubation time intervals using a random subset of the original samples that were used to assess first-pass success by modality. Without a complete analysis of the entire database, the selected sample may not fully represent the overall trends. Time intervals were manually recorded by human reviewers, which introduced the potential of inter-observer variability and potential timing inconsistencies, particularly when identifying precise start and end points of the intubation process. As a result, the conclusions regarding intubation time may be limited, and the overall generalizability may be constrained. Finally, although most intubations were performed by emergency medicine residents or attendings, a small proportion may have been completed by off-service residents or learners under supervision. These cases were not explicitly identified, which may have introduced a minor degree of uncertainty regarding operator background.

## CONCLUSION

The malleable stylet demonstrated the highest first-pass success rate and the most consistent intubation times, while the bougie had the longest times and lowest success rate in our ED. Physician preferences also favored the malleable stylet. First-pass success rates and intubation times vary depending on an institution’s familiarity with specific devices and clinician preferences. These factors should be considered when selecting intubation equipment to optimize airway management outcomes or facilitate training.

## Figures and Tables

**Figure 1 f1-wjem-26-1374:**
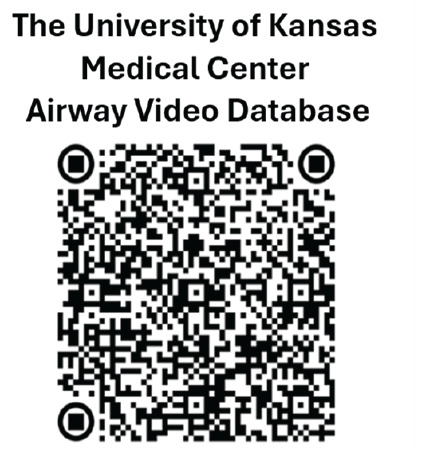
Quick response link to University of Kansas Medical Center airway video database, which was used to evaluate intubation success rates.

**Table 1 t1-wjem-26-1374:** This table displays the success rates of first-pass intubation attempts stratified by the type of stylet used. For each device type, the number and percentage of successful first attempts and those requiring multiple attempts are shown with 95% CIs and the total number and proportion of intubations performed using each device. Notably, the malleable stylet had the highest first-pass success rate (94.9%), while the bougie had the lowest (88.2%). A chi-square test was used to assess differences in success rates among devices.

First-pass Success by Device Type			
Stylet Type	First Attempt, n (%) 95% CI	Multiple Attempts, n (%) 95% CI	Total, n (%)
Hyperangulated	137 (93.2%) .89 – .97	10 (6.8%) .03 – .11	147 (25.57%)
Bougie	239 (88.2%) .84 – .92	32 (11.8%) .08 – .16	271 (47.13%)
Malleable	149 (94.9%) .92 – .98	8 (5.1%) .02 – .08	157 (27.3%)
Total	525 (91.3%) .89 – .93	50 (8.7%) .07 – .11	575 (100%)

χ^2^=6.53, P = .04.			

**Table 2 t2-wjem-26-1374:** Intubation time intervals for three device types: hyperangulated, bougie, and malleable. Time intervals were recorded for a subset of 70 intubations. For each device, the table shows the number of cases, percentage of total, minimum, median, mean, SD, maximum duration, and interquartile range (in seconds) required to complete intubation. We also report 95% CIs for median times tested. Ranked sum tests were used to compare time distributions across devices. The bougie group had the highest median and maximum times, while the malleable group had the shortest minimum time.

Intubation Time Intervals									
Intubation Device	*n*	Percentage	Min	Median	Mean	SD	95% CI for median	Max	IQR
Hyperangulated	23	32.86	16	31.00	33.61	11.00	28.85 – 38.37	54	18
Bougie	26	37.14	16	40.50	49.15	23.08	39.83 – 58.48	93	45
Malleable	21	30.00	13	31.00	33.81	16.40	26.34 – 41.28	72	18
Total	70	100	13	35.5	39.44	19.14	34.88 – 44.01	93	22

χ^2^=8.2019, P = .02.									

*Min*, minimum; *Max*, maximum; *IQR*, interquartile range.
